# Inhibitory Effect of Flower-Shaped Zinc Oxide Nanostructures on the Growth and Aflatoxin Production of a Highly Toxigenic Strain of *Aspergillus flavus* Link

**DOI:** 10.3390/ma11081265

**Published:** 2018-07-24

**Authors:** David Hernández-Meléndez, Enrique Salas-Téllez, Anai Zavala-Franco, Guillermo Téllez, Abraham Méndez-Albores, Alma Vázquez-Durán

**Affiliations:** 1 UNAM–FESC, Campus 4, Multidisciplinary Research Unit L14-Annex 1 (Materials Science and Technology), Cuautitlan Izcalli 54714, Mexico; david.hm.bqd@gmail.com; 2 UNAM–FESC, Campus 4, Multidisciplinary Research Unit L17 (Microbiology and Mycology), Cuautitlan Izcalli 54714, Mexico; satenrique@gmail.com; 3 Center for Research and Advanced Studies of the National Polytechnic Institute (CINVESTAV-IPN), Libramiento Norponiente 2000, Fraccionamiento Real de Juriquilla, Queretaro 76230, Mexico; anai.zavala@cinvestav.mx; 4 Department of Poultry Science, University of Arkansas, Fayetteville, AR 72701, USA; gtellez@uark.edu

**Keywords:** ZnO nanostructures, *Aspergillus flavus*, antifungal, anti-aflatoxigenic activity

## Abstract

Flower-shaped zinc oxide (ZnO) nanostructures were prepared via a simple aqueous precipitation strategy at room temperature. The as-grown nanostructures were characterized by UV–vis spectroscopy, UV–vis diffuse reflectance spectroscopy (DRS), spectrofluorometry, Fourier transform infrared (FTIR) spectroscopy with attenuated total reflection (ATR), X-ray diffraction (XRD), and field emission scanning electron microscopy (FESEM). The antifungal and anti-aflatoxigenic activities of the ZnO nanostructures were further investigated using a highly toxigenic strain of *Aspergillus flavus* Link under in vitro and in situ conditions. The results showed that the *A. flavus* isolate was inhibited to various extents by different concentrations of ZnO nanostructures, but the best inhibitions occurred at 1.25, 2.5, and 5 mM in the culture media. At these concentrations, suppression of aflatoxin biosynthesis (99.7%) was also observed. Moreover, a reasonable reduction in the aflatoxin content (69%) was observed in maize grains treated with the lowest ZnO concentration that exhibited the strongest inhibitory activity in the liquid media. SEM micrographs clearly indicate multiple degenerative alterations in fungal morphology after treatment with ZnO such as damage of the tubular filaments, loss of hyphae shape, as well as hyphae rupture. These results suggest that flower-shaped ZnO nanostructures exhibit strong antifungal and anti-aflatoxigenic activity with potential applications in the agro-food system.

## 1. Introduction

Transition metal-based oxides are among the most produced nanomaterials due to their multipurpose applications in chemistry, biology, medicine, biotechnology, molecular engineering, and physics. In recent times, dimensional nanostructured materials have attracted special attention; for that reason, considerable effort has been made to develop controlled synthesis protocols, since the properties and potential applications depend on their shape and size [1]. Zinc oxide (ZnO) is an important multifunctional, intrinsic II–VI semiconductor material, with a wide band gap (3.37 eV), large exciton binding energy (60 meV), high transmission coefficient, high chemical stability, and low threshold intensity [2,3]. Being an n-type semiconductor, the electron mobility in ZnO nanomaterials is enhanced [4]. ZnO (21 CFR 182.8991) is considered as a GRAS (generally recognize as a safe) substance by the United States Food and Drug Administration. At present, with the development of material science, it is expected that ZnO has further applications in many aspects of food and agriculture.

Mexico, with an estimated population of 124.7 million people, has the highest world per capita consumption of maize. In 2017, national maize production was 21.5 million metric tons [5]. Regrettably, maize is contaminated by certain fungal species, among them *Aspergillus flavus* Link. The toxins synthesized by *A. flavus* are named aflatoxins (AF), and include four major structures: AFB_1_, AFB_2_, AFG_1_, and AFG_2_ [6]. Aflatoxins represent a public health problem, since toxins could be synthesized both in the field as well as in the storage [7]. AFB_1_ has strong carcinogenicity potential; consequently, it has been classified by the International Agency for Research on Cancer as a human carcinogen, Group 1 [8].

The best approach to control aflatoxin contamination in maize is to prevent aflatoxin formation. In the field, the aflatoxin production threat can be diminished by the use of some agricultural practices that limit the establishment of the fungus on the maize cob [9]. In the postharvest system, an appropriate drying process can mitigate the aflatoxin formation during grain storage; however, developing countries lack access to postharvest technology. Moreover, fungi can be controlled by organic synthetic fungicide application, but their control is difficult, due to resistance to many traditional antifungal agents such as benzimidazoles, demethylation inhibitors, Qo respiration inhibitors, and dicarboximides [10]. To overcome this situation, it is imperative to explore other alternatives. Although antifungal activity of ZnO nanoparticles has been widely studied, just few articles have been published on their antifungal and anti-aflatoxigenic activity against *A. flavus* Link [11,12,13,14]. However, no attempt has yet been made to evaluate the inhibitory effect of nanostructured ZnO against a highly toxigenic strain of *A. flavus*. Therefore, the objectives of the present research were to synthesize and characterize flower-shaped ZnO nanostructures, and evaluate their antifungal and anti-aflatoxigenic effectiveness against a highly toxigenic strain of *A. flavus* Link in liquid media and on maize grains.

## 2. Materials and Methods

### 2.1. Safety Measures in Laboratory

Sodium hypochlorite 6% (*w*/*v*) was used to surface-disinfect equipment and working areas. Wastes containing fungal spores and mycotoxins were first steam sterilized and then immersed in the sodium hypochlorite solution overnight.

### 2.2. Chemical Compounds

Zinc nitrate hexahydrate (Zn(NO_3_)_2_·6H_2_O; 98% purity; CAS number 10196-18-6), potassium hydroxide (KOH; 90% purity; CAS number 1310-58-3), glutaraldehyde solution (OHC(CH_2_)_3_CHO; 50% in H_2_O; CAS number 111-30-8), tween-80 (CAS number 9005-65-6), methanol (CH_3_OH, CAS number 67-56-1), methanol HPLC grade (CH_3_OH, CAS number 67-56-1), sodium chloride (NaCl, CAS number 7647-14-5), and Sabouraud dextrose broth (SDB) were obtained from Merck KGaA (Darmstadt, Germany). Dehydrated culture media: potato dextrose agar (PDA) was procured from BD Bioxon (Becton Dickinson de Mexico SA de CV, Estado de Mexico, Mexico). Bromine solution (0.03%) was purchased from Vicam (Milford, MA, USA). All solutions were prepared using deionized water.

### 2.3. Synthesis and Characterization of ZnO Nanostructures

Flower-shaped ZnO nanostructures were engineered at room temperature as follows: 100 mL aqueous solution of Zn(NO_3_)_2_·6H_2_O (0.4 M) was vigorously stirred for 20 min in a 250 mL flat bottom flask. Afterwards, KOH (2 M) solution was added to the zinc solution to rise the pH value to 12. After completion of the reaction (2 h), the resultant suspension was aged for 12 h. The white suspension was centrifuged (3200× *g*, 5 min), and the precipitate was exhaustively washed with deionized water to remove the byproducts. Finally, the sample was oven dried at 120 °C for 2 h and annealed at 600 °C for 1 h, in air atmosphere.

ZnO nanostructures were further characterized by UV–vis absorption (Agilent Technologies, Santa Clara, CA, USA), diffuse reflectance spectroscopy (DRS, Perkin Elmer, Waltham, MA, USA), fluorescence (Perkin Elmer, Waltham, MA, USA), and Fourier transform infrared spectroscopy with attenuated total reflection (FTIR-ATR, Perkin Elmer, Waltham, MA, USA) according to the methodologies proposed by Estrada-Urbina et al. [15]. The X-ray measurements were recorded with a 2100-Rigaku diffractometer (Rigaku Co., Tokyo, Japan). CuK*_α_* radiation and a fixed power source of 30 kV and 20 mA were used. The X-ray diffraction patterns were obtained in the region of 2*θ* from 25° to 75°, using a 0.02° step size. The size of the crystallite was calculated using the Scherrer´s equation [16].

(1) $$D=\frac{k\lambda }{\beta \;cos\theta }$$

where *D* is the average crystallite size; *k* is the Scherrer coefficient (0.9); *λ* is the X-ray wave length (1.5405 Å); *β* is the full width at half maximum in radians; and *θ* is Bragg´s angle (2*θ*). Finally, the morphology and size of the ZnO nanostructures was studied using the field emission scanning electron microscopy (FESEM) model JSM-7610F (JEOL, Tokyo, Japan). Microscopy analysis was performed at 10,000× and 100,000× with an accelerating voltage of 2 kV.

### 2.4. Laboratory Experiments

#### 2.4.1. Fungal Isolate

The highly toxigenic strain of *Aspergillus flavus* Link (UNIGRAS-1231, Culture Collection of the Grain and Seed Research Unit of the National Autonomous University of Mexico) was grown in potato-dextrose-agar (PDA) medium for 7 d at 25 °C. This fungus mainly produces AFB_1_ [17]. A sterile-water conidia suspension containing 0.5% Tween-80 was prepared with approximately 4 × 10^6^ spores/mL.

#### 2.4.2. Effect of Nanostructured ZnO on the Growth of the Fungus and Aflatoxin Production in Liquid Media

For the experiment, 125 µL of the conidia suspension (500,000 spores) were added to 250 mL round bottom flasks containing 100 mL of Sabouraud dextrose broth (SDB). Different concentrations of the ZnO nanostructures (0.3125, 0.625, 1.25, 2.5, and 5 mM) were added to the flasks and cultures were incubated under agitation at 170 rpm using an orbital shaker (PSU-10i, Boeco, Hamburg, Germany) for 3 d at 25 °C. At the end of the incubation period, the content of each flask was filtered through a Whatman no. 4 filter paper and subjected to AFB_1_ determination. The vegetative mycelium was used for dried weight estimation; the mycelium was dried at 70 °C for 72 h, till their weights remains constant. For further detection on the effects of nanostructured ZnO on the fungus, scanning electron microscopy (SEM) was used. For this purpose, the mycelia was fixed for 3 h in 2.5% (v/v) glutaraldehyde (100 mM phosphate buffer solution, pH 7.2), oven dried at 40 °C for 24 h, and coated using a gold-sputter (Denton Vacuum Inc., Desk V HP, Moorestown, NJ, USA) for 5 min. Microscopy analysis was performed using a SEM (JEOL, JSM-6012LA, Tokyo, Japan) at 500×, 1000×, and 5000× with an accelerating voltage of 15 kV.

#### 2.4.3. Effect of Nanostructured ZnO on the Growth of the Fungus and Aflatoxin Production in Maize Grains

The nanostructured ZnO concentration that exhibited the strongest inhibitory activity in the liquid media experiment was further tested on maize grains. The fungal inoculation procedure was followed in accordance to the methodology suggested by Méndez-Albores et al. [18]. Regular maize of the commercial hybrid AS-900 (Aspros Comercial, Guanajuato, Mexico) grown and harvested in 2017 at Celaya-Guanajuato, Mexico, with 11.5% moisture content (MC) was utilized. Experimental units containing 100 g of maize (five replicates of each treatment) were adjusted to 18% MC and stored in 500 mL flasks resistant to steam sterilization. The water that was added to reach the desired MC served as a vehicle for the inoculum (5000 spores per gram of maize). To minimize the loss of MC from the maize grains, flasks were protected with polyethylene film. Ten perforations with a sterile pin were made to each film to prevent CO_2_ accumulation. Flasks were incubated at 27 °C for 7 days. This short-term storage period was chosen, due to the highly favorable temperature and humidity conditions for the development of the toxigenic fungus in the maize grains. At the end of the incubation, experimental units were sterilized using ethylene oxide gas at a concentration of 1000 mg/L during 5 h [19]. Finally, the percentage of grains invaded by the fungus as well as the aflatoxin content was determined.

#### 2.4.4. Aflatoxin Assay

Aflatoxin analysis was carried out following the recommendations of the 991.31 AOAC method [20], with immunoaffinity columns for AFB_1_ (VICAM, Milford, MA, USA). Samples (culture media or maize grains) were blended with 100 mL methanol–water (80:20 v/v) and 5 g of NaCl, and filtered through a Whatman no. 1 filter paper. Then, 10 mL of filtrate were diluted with 40 mL of deionized water, and the dilution filtered again with a micro-fiber filter. Subsequently, 10 mL of the diluted sample were passed through an immunoaffinity column (Aflatest-P; VICAM Science Technology, Watertown, MA, USA), which was then washed twice with 10 mL of deionized water. Aflatoxins were eluted from the immunoaffinity columns by passing 1 mL of methanol HPLC grade, and the toxins were collected in a fluorometer glass cuvette. Finally, 1 mL of 0.003% bromine solution was added to the elution and aflatoxins were quantified after 60 seconds in a fluorometer VICAM Series-4Ex (VICAM Source Scientific, Irvine, CA, USA). The limit of detection for aflatoxins using immunoaffinity columns is about 0.5 ng/g.

### 2.5. Statistical Analysis

The experiment was carried out under a completely randomized design. The results were analyzed via analysis of variance (ANOVA) utilizing the Statistical Analysis System [21]. Mean values were separated with the Dunnett procedure using a significance value of *p* < 0.05.

## 3. Results and Discussion

### 3.1. Characterization of Nanostructured ZnO

#### 3.1.1. Optical Properties

**Figure 1:**
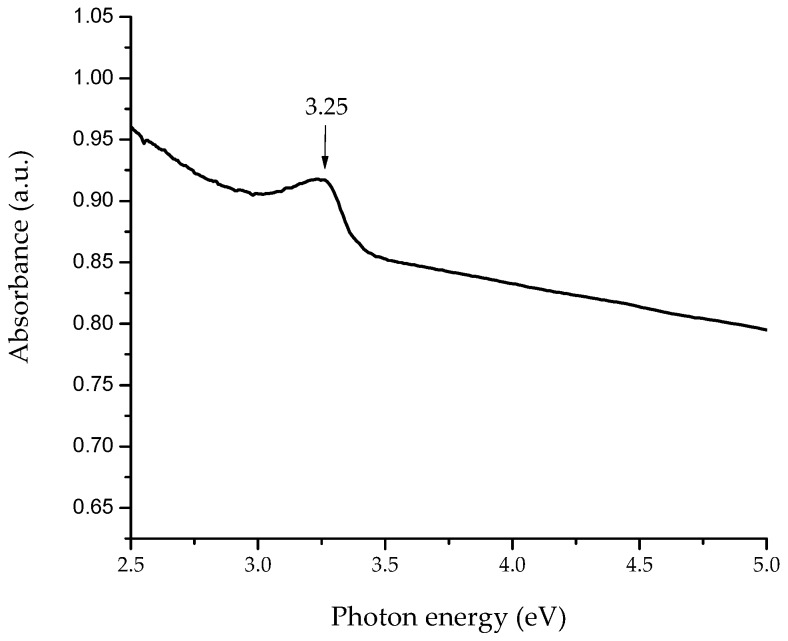
UV–vis absorption spectrum of synthesized flower-shaped zinc oxide (ZnO) nanostructures.

The UV–vis absorption spectrum of flower-shaped ZnO nanostructures is shown in Figure 1. The spectrum revealed the distinctive absorption peak (3.25 eV), corresponding to the optical band gap of ZnO, which is around 3.24 eV [22]. The spectrum have two components: (1) the optical absorption due to electronic transitions in the nanostructured ZnO, and (2) the dispersed light due to the scattering phenomenon, taken as absorbed light by the spectrophotometer [23].

**Figure 2:**
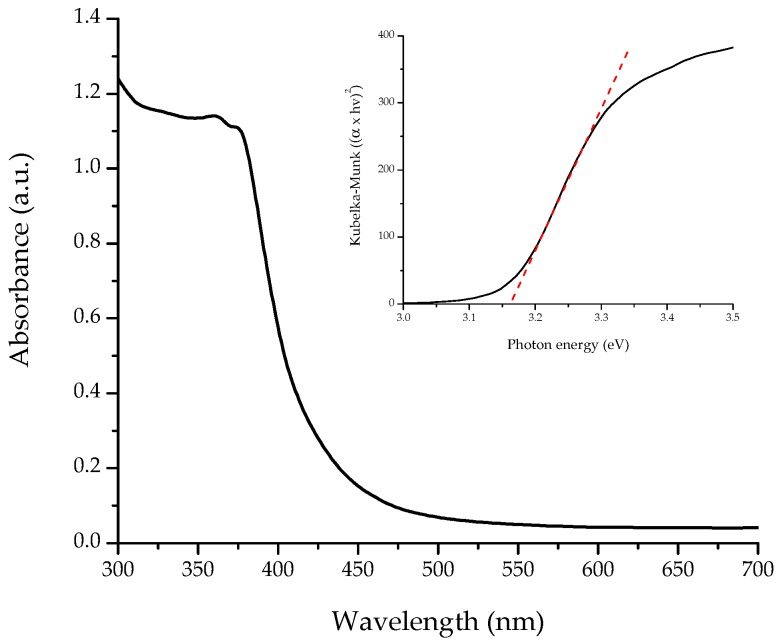
UV–vis diffuse reflectance spectrum of synthesized flower-shaped ZnO nanostructures. The inset shows their corresponding Kubelka-Munk transformed reflectance spectrum.

To avoid certain difficulties in obtaining the true optical band gap value of ZnO from UV–vis in dispersed samples, DRS measurements of powders were performed. As seen in Figure 2, the UV–vis spectrum showed a strong absorption edge at 400 nm, related to the electron transitions from the valence band to the conduction band (O_2p_ → Zn_3d_). The DRS spectrum of the flower-shaped ZnO nanostructures after Kubelka-Munk treatment is shown in the inset of Figure 2. The intersection between the linear fit and the *x* axis (photon energy) gives the optical band gap value. As a result, the direct band gap of the nanostructured ZnO was 3.17 eV. Yu et al. [24] reported a band gap value of 3.16 eV for hexagonal phase ZnO rods with average diameter of 2 µm and length of 7 µm. Compared with bulk ZnO (3.37 eV), the absorption edge of the flower-shaped ZnO nanostructures is red-shifted. This might be due to changes in morphology, particle size, and surface microstructures [25].

**Figure 3:**
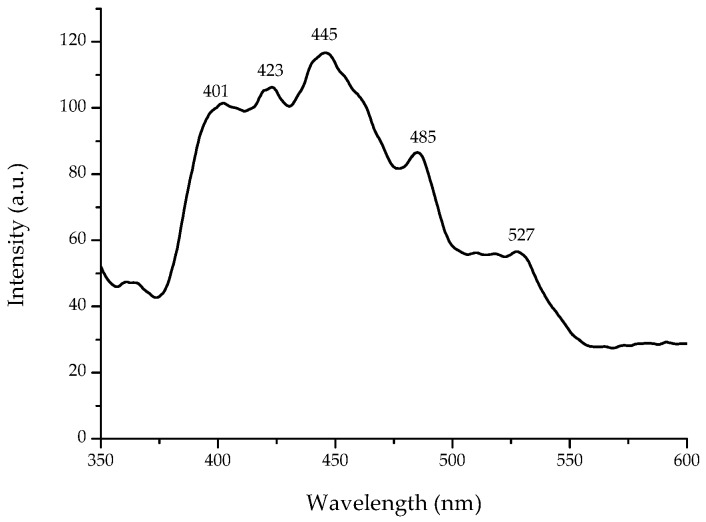
Room temperature fluorescence spectrum of synthesized flower-shaped ZnO nanostructures.

Fluorescence spectroscopy is often used to investigate certain optical properties of semiconductors. Figure 3 shows the fluorescence spectrum at room-temperature of the flower-shaped ZnO nanostructures with an excitation wavelength of 325 nm. Five emission bands, including a strong ultraviolet emission at 401 nm (3.09 eV), were observed. Irimpan et al. [26] reported that the ultraviolet emission of ZnO has been related to the band gap fluorescence of clusters of different sizes [26]. In addition, the formation of Zn interstitials defects are associated with the blue emission at 423 nm (2.93 eV), and the generation of oxygen vacancy defects are assigned to the blue emission at 445 nm (2.79 eV) and blue-green emission at 485 nm (2.56 eV), respectively. Moreover, antisite defects originate the green fluorescence at 527 nm (2.35 eV) [15]. According to Vempati et al. [27], there are numerous energy levels due to defects such as zinc and oxygen vacancies and/or interstitials.

#### 3.1.2. FTIR Analysis

**Figure 4:**
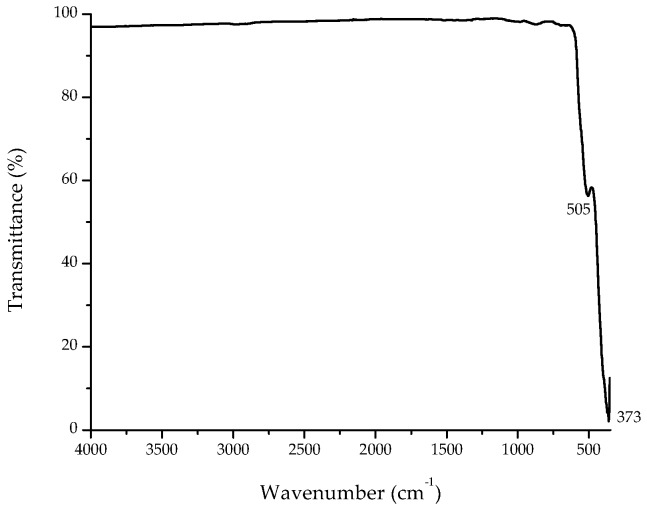
Fourier transform infrared spectroscopy with attenuated total reflection (FTIR-ATR) spectrum of synthesized flower-shaped ZnO nanostructures.

The composition and quality of the flower-shaped ZnO nanostructures was analyzed by means of the FTIR-ATR spectroscopy technique. Figure 4 shows the spectrum, which was collected in the range of 350–4000 cm^−1^. Annealing in air atmosphere at 600 °C for 1 h significantly reduces the bands intensities in the region of 2800–3600 cm^−1^, due to the removal of water molecules and the convertion of Zn(OH)_2_ to ZnO. In addition, the intensity of the impurities related FTIR signatures (1400–1600 cm^−1^) decreased significantly as a consequence of the decomposition of organic complexes. However, two significant bands were observed at 505 cm^−1^ and 373 cm^−1^ (Figure 4). While the band at 505 cm^−1^ corresponds to oxygen deficiency and/or oxygen vacancy defect complex in ZnO [28], the broad band at 373 cm^−1^ may be attributed to the Zn–O stretching vibration mode [25]. 

#### 3.1.3. X-ray Analysis

**Figure 5:**
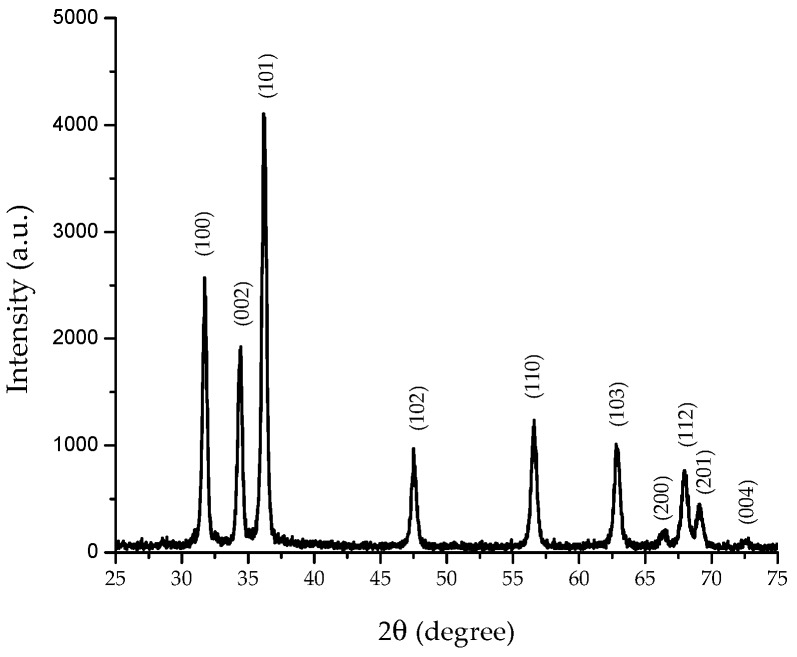
Representative X-ray diffraction pattern of synthesized flower-shaped ZnO nanostructures.

XRD studies provide information about crystal structure, orientation, crystallite size, phase, and lattice parameters, among others. Figure 5 shows the XRD pattern of the synthesized ZnO nanostructures. All diffraction peaks for (1 0 0), (0 0 2), (1 0 1), (1 0 2), (1 1 0), (1 0 3), (2 0 0), (1 1 2), (2 0 1), and (0 0 4) planes are related to the formation of hexagonal (wurzite) structure of ZnO, which is in accordance to the standard card JCPDS 36-1451. No other diffraction peaks from byproducts were observed.

In addition, the broadening of the XRD peaks indicates that the crystallite size of the nanostructured ZnO is in the nanometer range. The crystallite size was determined using the Scherrer equation. The (1 0 0) plane was selected for calculation, and the average crystallite size was 42.0 ± 0.8 nm. The calculated lattice parameters were a = b = 3.254 Å, and c = 5.207 Å (c/a = 1.6). Chand et al. [3] reported similar lattice constant values (a = 3.249 Å, and c = 5.207 Å) for ZnO nanostructures synthetized at pH 12, which is consistent with our findings. 

#### 3.1.4. Morphology and Size of Nanostructured ZnO

**Figure 6:**
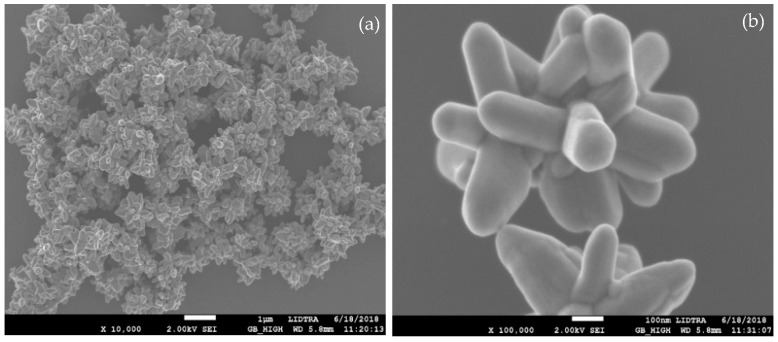
(**a**) Field emission scanning electron microscopy (FESEM) image of synthesized flower-shaped ZnO nanostructures; and (**b**) a high magnification image of a single flower.

Figure 6 shows the field emission scanning electron microscopy (FESEM) image of the nanostructured ZnO. The image reveals that large-scale 3D flower-like ZnO is formed, and that most of the structures are in a uniform fashion, with diameters in the range of 700–800 nm. The flower-shaped ZnO nanostructures are composed of structurally arranged flattened tipped hexagonal nanorods with diameters and lengths of around 120 nm and 280 nm, respectively.

It can be seen that all hexagonal nanorods are joined together through their bases forming a flower-shaped morphology. It is well known that the shape and the size of the obtained ZnO depend strongly on the reaction conditions. In the literature, both precursors (zinc nitrate and potassium hydroxide) have been used to synthesize mainly spherical nanoparticles [29,30]. However, in this research, by using different reaction conditions, flower-shaped ZnO nanostructures were obtained. Besides, the reaction was carried out without the presence of surfactants. Li et al. [31] reported that star-like structures with 3, 4, 5, 6, or 7 branches, or flower-like particles with different numbers and shapes of leaves were synthesized at zinc acetate concentrations of 0.1 M and above.

### 3.2. Antifungal and Anti-Aflatoxigenic Studies

#### 3.2.1. Antifungal Activity (Liquid Media)

**Figure 7:**
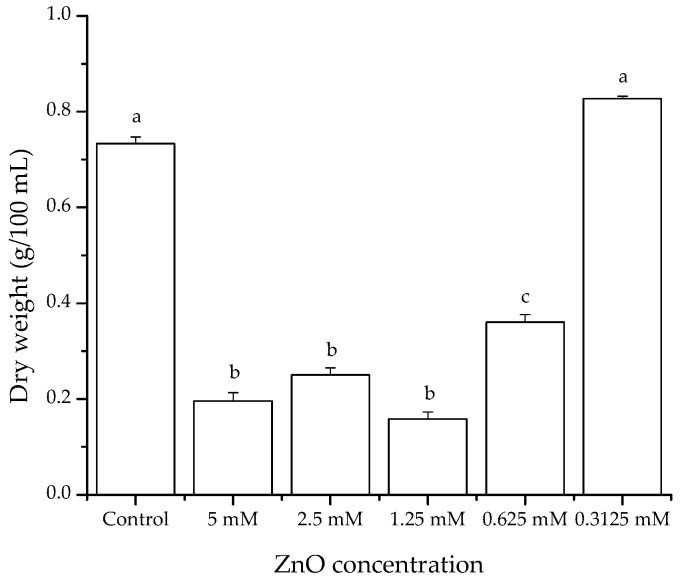
Effect of nanostructured ZnO on mycelial growth of *Aspergillus flavus* in culture media. Mean values ± standard error of three independent experiments. Bars not sharing a common superscript differ significantly (Dunnett < 0.05).

Data indicate that nanostructured ZnO at a concentration of 0.3125 mM showed no significant differences in mycelial dry weight, presenting average values similar to the control (0.78 g/100 mL culture media). It has been reported that low concentrations of solubilized Zn^2+^ can trigger a relatively high tolerance in microorganisms. However, a notable reduction in mycelial production of *A. flavus* was observed at concentrations of 1.25, 2.5, and 5 mM ZnO, showing reductions up to 78% of the control level. ZnO at 0.625 mM has the lowest reduction in mycelial growth, reaching values up to 51% (Figure 7). 

Our results are in accordance to those of Jasim [32], who reported a reduced mycelial production (60%) of the fungus *A. fumigatus* in culture media when using 6 mM of ZnO nanoparticles (<50 nm). In another study in culture media, reductions up to 79% in mycelial production of *A. flavus* and *A. parasiticus* were reported using 0.325–1.5 mg/mL of *Agave asperrima* and *Agave striata* extracts [33]. Data are consistent with our findings.

#### 3.2.2. Anti-Aflatoxigenic Activity (Liquid Media)

**Figure 8:**
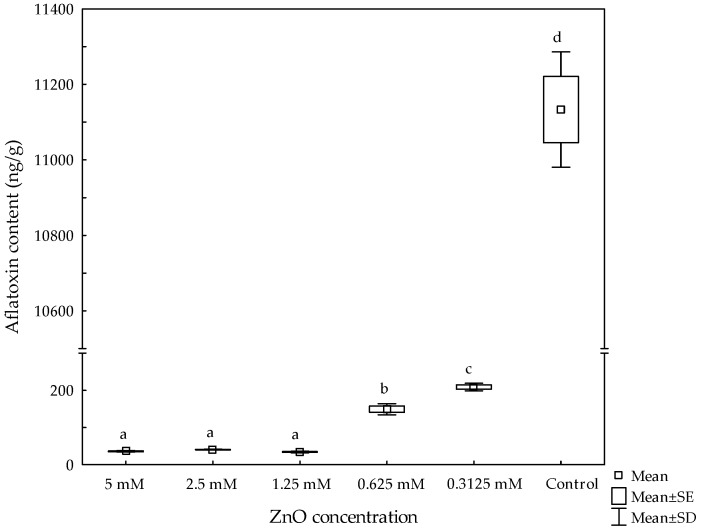
Effect of nanostructured ZnO on aflatoxin production in culture media. Boxes and whiskers not sharing a common superscript differ significantly (Dunnett < 0.05).

Regarding aflatoxin biosynthesis, the mycotoxin content increased as the ZnO concentration decreased in the culture media (Figure 8). The aflatoxin content in the control was 11,133 ng/g. At concentrations of 1.25, 2.5, and 5 mM ZnO, the aflatoxin content decreased 99.7%, the average aflatoxin content in these experimental units was about 37 ng/g. However, a 98.7% and 98.1% decrease was observed at 0.625 mM and 0.3125 mM ZnO, reaching aflatoxin values of 149 ng/g and 209 ng/g, respectively. In this research, in just 3 d, the *A. flavus* isolate was able to produce extremely high quantities of aflatoxins. 

When *A. parasiticus* PTCC5280 was inoculated (10^6^ conidia/mL) to potato-dextrose broth (PDB) for 7 d, an aflatoxin value of 70 ng/g was attained [34]. In the study, silver nanoparticles (90 µg/mL) completely inhibited the aflatoxin production. Furthermore, Al-Othman et al. [35] reported that five *A. flavus* isolates were inhibited to various extents by different concentrations of silver nanoparticles using SMKY liquid medium (sucrose, magnesium sulfate, potassium nitrate, and yeast extract). At a concentration of 150 µg/mL, 100% inhibition of aflatoxin production was reached. The aflatoxin values in control samples incubated during 20 d ranged from 18.6 to 69.3 ng/g. In another study, Hassan et al. [11] found that 8 µg/mL of ZnO nanoparticles was an effective concentration to inhibit the production of aflatoxins by an *A. flavus* strain inoculated in YES (yeast extract and sucrose) broth for 20 d. Control samples produced an aflatoxin content of 20 ng/g. The inhibitory effect of ZnO on aflatoxin production varies considerably depending on several parameters, such as fungal isolate, spore load, culture media, incubation time, temperature, as well as the nanomaterial-type being evaluated.

#### 3.2.3. Structural Examination of the Fungus

**Figure 9:**
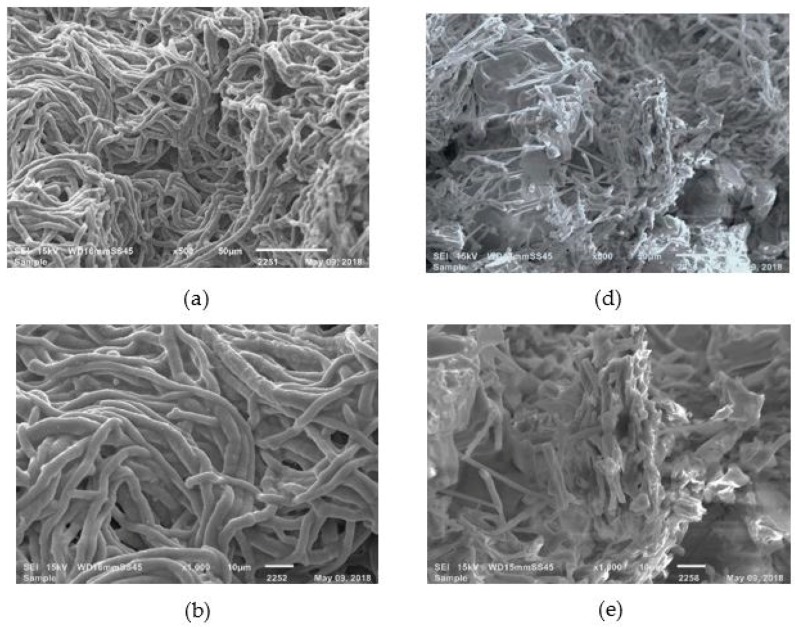

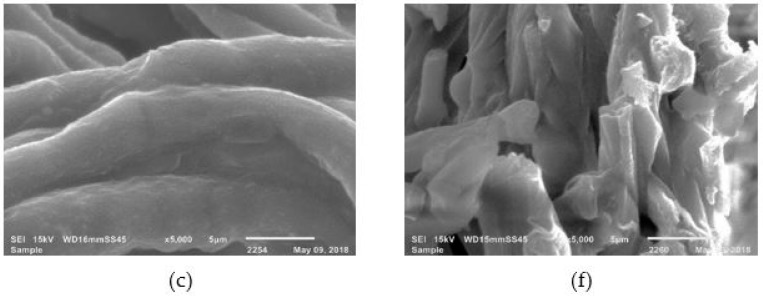
Scanning electron microscopy of *Aspergillus flavus* mycelium: (**a**–**c**) without treatment; (**d**–**f**) with nanostructured ZnO treatment at 1.25 mM. Profiles c,f are enlargements of b,e, respectively.

To determine the effect of ZnO on the growth of *A. flavus* and its ability to synthesize aflatoxins, SEM analysis was conducted evaluate the structural changes in the mycelium. SEM micrographs clearly indicate multiple degenerative alterations in morphology after treatment with ZnO at 1.25 mM (Figure 9d–f), compared with the control group (Figure 9a–c). In general, untreated samples presented unicellular filaments with their typical structure with a smooth surface. However, after ZnO treatment, the tubular filaments showed damage such as deformation in mycelial growth, loss of hyphae shape and smoothness, as well as hyphae rupture. Cell membrane rupture probably results in reduction of the enzymatic activity of the fungus, leading to a significant reduction in mycelial dry weight and consequently, a possible interruption in the aflatoxin biosynthetic process.

It is well known that ZnO is moderately soluble and can release zinc ions (Zn^2+^) in solution. There are two main approaches on the antifungal activity of ZnO: reactive oxygen species (ROS) generation and the release of Zn^2+^ in the medium. Released Zn^2+^ has significant effect in the amino acid metabolism and enzyme system disruption. Elevated intracellular Zn^2+^ also induced disruption of mitochondrial function [36,37], and triggered ROS generation [38]. Song et al. [39] evaluated the role of dissolved Zn^2+^ ions and ROS in the cytotoxicity of ZnO nanoparticles in the mouse macrophage Ana-1 cells. Results showed that when the concentration of ZnO nanoparticles exceeded 40 µg/mL, the dissolved Zn^2+^ concentration tended to equilibrium (10 µg/mL) in the complete cell medium. At concentrations of ZnO up to 100 µg/mL, the dissolved Zn^2+^ concentration of fine ZnO, 100, 30, and 10–30 nm ZnO particles were 9.9, 9.7, 9.6, and 10.3 µg/mL, respectively. Authors conclude that Zn^2+^ ions were responsible for the toxic effect of ZnO nanoparticles. ROS generation of nano-ZnO was not enough to cause cytotoxicity. 

In this research, the antifungal and anti-aflatoxigenic effects of ZnO nanostructures were probably due to the release of Zn^2+^ ions into the SDB. Taking into account that the pH value of the broth was approximately 5.9, and according to the phase stability diagram for the ZnO(s)-H_2_O [40,41], the soluble ionic form of ZnO is mainly Zn^2+^, which is predominant under slightly acidic conditions of pH < 6.0 [42]. 

#### 3.2.4. Fungal Invasion and Aflatoxin Production in Maize Grains

The inoculated maize allowed the proliferation of *A. flavus* and the subsequent aflatoxin production. The aflatoxin production in maize was very low compared to liquid media. The results indicate that ZnO at 100 µg/g of maize was almost effective under the experimental conditions tested. As expected, the only fungus isolated was *A. flavus*, whose presence was abundant in the control grain, presenting a 67% fungal invasion. The high incidence of *A. flavus* agrees with the production of aflatoxins (45 ng/g). However, maize treated with ZnO, presented a moderate fungal invasion (30%), and a relatively low production of aflatoxins (14 ng/g). Such aflatoxin content was below the maximum tolerance (20 ng/g) permissible in Mexico for aflatoxin contamination in maize intended for human consumption [43]. Nabawy et al. [13] reported aflatoxin levels of 46 ng/g in yellow maize, and values of 12 ng/g in maize treated with 100 µg/mL spherical ZnO nanoparticles (100 nm). The experimental conditions tested were: *A. flavus*, isolates recovered from samples of animal feed; spore load, 10^5^ spores/mL; incubation temperature, 30 °C; incubation time, 1 month. To the best of our knowledge, the present study is the first one to demonstrate that 3D flower-like ZnO nanostructures—composed of 1D nanorods—possess significant antifungal and anti-aflatoxigenic activities against a highly toxigenic strain of *Aspergillus flavus* Link under in vitro and in situ conditions.

## 4. Conclusions

Flower-shaped ZnO nanostructures have significant antifungal and anti-aflatoxigenic activity against a highly toxigenic strain of *Aspergillus flavus* Link. ZnO nanostructures at a concentration of 1.25 mM significantly inhibited the growth and aflatoxin production of the fungus in culture media. Furthermore, maize treated with ZnO (100 µg/g of maize), presented a moderate fungal invasion and a relatively low production of aflatoxins. Therefore, the application of ZnO nanostructures could be recommended as an effective fungicide against the major fungal pathogen of maize.
